# Editorial: Innovative applications of hyperspectral imaging technology in horticultural plants

**DOI:** 10.3389/fpls.2026.1939703

**Published:** 2026-07-30

**Authors:** Weijie Lan, Longguo Wu, Houcheng Liu, Juan Francisco García Martín

**Affiliations:** 1 College of Food Science and Technology, Nanjing Agricultural University, Nanjing, Jiangsu, China; 2 School of Enology and Horticulture, Ningxia University, Yinchuan, China; 3 College of Horticulture, South China Agricultural University, Guangzhou, China; 4 Departamento de Ingeniería Química, Facultad de Química, Universidad de Sevilla, Seville, Spain

**Keywords:** cultivation environment optimization, growth status monitoring, horticultural plants, hyperspectral imaging technology, quality evaluation, technology integration, variety identification

## Introduction

Against a backdrop of growing demand for natural, chemical-free high-quality food, coupled with increasingly varied growing conditions due to climate change, the application of hyperspectral imaging (HSI) technology to horticultural crops is gradually gaining attention. This is because it is regarded as an excellent tool that not only enables the accurate monitoring and analysis of various conditions affecting these crops, but also contributes to more sustainable horticultural production.

This editorial provides an in-depth discussion of how this technology breaks through traditional limitations and brings about new changes in the horticultural field. Specifically, through research and elaboration, it reveals how HSI technology can accurately monitor and deeply analyze the growth status, nutritional levels, occurrence of diseases and pests, etc. of horticultural plants. This provides a scientific basis for the cultivation and management of horticultural plants and helps to achieve sustainable horticultural production. Additionally, integrated application with other technologies such as machine learning and unmanned aerial vehicles is also important in expanding the scope and depth of its application in the field of horticultural plants. Furthermore, it will promote the modern development of the entire horticultural industry to meet consumers’ demands for high-quality and safe horticultural products.

In summary, this Research Topic focused on four key areas for the implementation of HSI:

1. Monitoring the growth status of horticultural plants.

Through in-depth analysis of the plant’s spectral information, problems that occur during the plant’s growth process can be detected in a timely manner, providing a basis for taking corresponding measures, thus ensuring the healthy growth of plants. As reported by Shoaib et al., HSI and multispectral imaging, together with new technologies in the fields of digital imaging, thermal imaging and optical filters, as well as advances in smartphone cameras, have made it possible to develop low-cost, field-ready platforms that enable real-time monitoring of plant stress. In their paper, these authors explored the potential for applying machine learning to multimodal images and smartphone-based solutions to provide affordable agricultural diagnostics, highlighting their limitations and prospects.

2. Quality and safety evaluation of horticultural plants.

Explore the principles and methods of using HSI to evaluate the quality of horticultural plant fruits (such as sweetness, acidity, firmness, etc.), and compare the advantages over traditional detection methods. For example, the soluble solids content is a standard parameter to assess the quality of pineapples (*Ananas comosus*) on an industrial scale, and it has a major influence on grading and sales. Yao et al. investigated the non-destructive determination of pineapple’ soluble solids content using 100 fruits from 5 pineapple cultivars and a HSI system that collects hyperspectral images in the 400–1700 nm range. The best mathematical model developed by the authors achieved R² of 0.9869 and RMSE of 0.125 Brix, highlighting four key wavelength ranges: 673–676 nm, 711–715 nm, 971–990 nm and 1357–1367 nm, and hence confirming that HSI enables accurate determination of the soluble solids content in pineapples. With regards to potential pollutants, microplastics pose an ever-increasing threat to global food security. Hence, the development of non-destructive methods for their early detection an urgent scientific priority. Concerning rice seedling, two novel, non-destructive, and interpretable frameworks have been developed for the early detection of microplastics (polyethylene terephthalate, polystyrene and polyvinyl chloride) in rice seedlings. Firstly, Wei et al. used two complementary spectroscopic techniques, namely visible/near-infrared HSI and synchrotron radiation-based Fourier transform infrared spectroscopy, thereby achieving a detection accuracy rate exceeding 93%. In addition, SHapley Additive exPlanations (SHAP) analysis of the model made it possible to identify the bands associated with chlorophyll, carotenoids, water content and cellulose. Secondly, Wei et al. developed another framework based on excitation-emission matrix fluorescence spectra combined with deep learning. Their optimal model achieved a classification accuracy of 100%. Furthermore, SHAP analysis of the model identified humic acid-like and marine humic acid-like components as the main factors contributing to the model.

3. Identification and classification of horticultural plant varieties.

Elaborate on how to obtain the unique spectral characteristics of different horticultural plant varieties through hyperspectral imaging, establish variety identification models, achieve rapid and accurate variety identification and classification, and its applications in aspects such as seed purity detection. For example, the quality of *Fritillariae cirrhosae bulbus* is influenced by its geographical origin and cultivation management. Han et al. integrated untargeted metabolomics, alkaloid quantification, mineral nutritional element analysis, and HSI features to systematically reveal the significant effects of geographical origin and agronomic practices on the metabolite composition, whilst establishing a multidimensional evaluation framework based on metabolism, components and the environment.

4. Optimizing the cultivation environment of horticultural plants.

Study how to use HSI to monitor the impacts of factors such as light, temperature, and soil fertility in the cultivation environment of horticultural plants on plant growth, providing data support and decision-making basis for precise cultivation. In this sense, Deng et al. mapped the spatial distribution of predicted sorghum yield, as well as spatial clustering and cyclic changes, in arid regions using HSI unmanned aerial vehicle coupled with stacking ensemble learning. Their data makes it possible to identify the critical growth stages that need to be monitored, thereby reducing data collection costs without compromising the high accuracy of the predictions. Furthermore, the authors generated high-resolution yield prediction maps featuring a comprehensive analysis of spatial patterns, with a view to supporting site-specific management decisions and precision sorghum irrigation planning.

## Conclusions and future perspectives

The contributions included in this Special Issue collectively demonstrate the broad potential of HSI for monitoring plant growth, evaluating product quality and safety, identifying varieties and geographical origins, and supporting precise cultivation. Despite the diversity of crops and application scenarios, these studies share several methodological features, including the extraction of informative spectral or spatial-spectral features, the integration of hyperspectral data with machine learning or deep learning, and the increasing use of multimodal sensing, interpretable artificial intelligence, and unmanned aerial vehicles. Together, these developments indicate that HSI is evolving from a laboratory-based analytical technique into a more comprehensive decision-support tool for horticultural production. Future research should prioritize the construction of larger, standardized, and multi-site datasets covering different cultivars, seasons, stress conditions, and cultivation systems. Finally, reducing the gap between laboratory and practical field deployment will require portable and low-cost sensors, automated calibration and preprocessing procedures, efficient feature extraction, lightweight predictive models, and real-time data-processing systems. Addressing these methodological and practical challenges will help transform HSI from a promising research technology into a reliable tool for sustainable, precise, and intelligent horticultural production.

